# Enhancing the Quality of Low-Salt Silver Carp (*Hypophthalmichthys molitrix*) Surimi Gel Using Psyllium Husk Powder: An Orthogonal Experimental Approach

**DOI:** 10.3390/gels10040247

**Published:** 2024-04-04

**Authors:** Muhammad Safeer Abbas, Lizhi Xia, Qiang Li, Yufeng Lu, Songkun Liu, Lin Lin, Jianfeng Lu

**Affiliations:** 1 School of Food and Biological Engineering, Hefei University of Technology, Hefei 230601, China; abbassafeer999@mail.hfut.edu.cn (M.S.A.); 2021171516@mail.hfut.edu.cn (L.X.); lq00742021@163.com (Q.L.); 13855526017@163.com (Y.L.); 2021171448@mail.hfut.edu.cn (S.L.); linlin@hfut.edu.cn (L.L.); 2 Engineering Research Center of Bio-Process, MOE, Hefei University of Technology, Hefei 230601, China; 3 Key Laboratory for Agricultural Products Processing of Anhui Province, Hefei University of Technology, Hefei 230601, China

**Keywords:** gel strength, water-holding capacity, orthogonal experiment, optimized gel, hardness, hydrophobic interactions, denser gel structure

## Abstract

Low-salt surimi production is crucial as it addresses health concerns related to sodium intake while maintaining the quality and shelf-life of seafood products. This research focused on optimizing the gelation conditions for silver carp surimi with the addition of psyllium husk powder at low salt concentrations (0.5% and 1%, *w*/*w*) to investigate the effects of psyllium husk powder concentration, temperature, and time on gel strength and water-holding capacity. The quality was assessed in terms of gel strength and water-holding capacity. Following a single-factor exploration, a three-level orthogonal experiment was designed to evaluate the influence of these three variables using a combined scoring system. Results indicated that psyllium husk powder levels between 0.1% and 0.3% (*w*/*w*) enhanced gel strength and water-holding capacity. The optimal conditions were identified as follows: 1% (*w*/*w*) NaCl with 0.2% (*w*/*w*) psyllium husk powder for 2.5 h at 35 °C, and 0.5% (*w*/*w*) NaCl with 0.3% (*w*/*w*) psyllium husk powder for 3 h at 35 °C. Texture profile analysis revealed that psyllium husk powder increased the hardness of the surimi gel, promoting myosin cross-linking and denser gel structure. Compared to traditional surimi gel, which relies on ionic bonds, the optimized gel showed higher levels of disulfide cross-linking and enhanced hydrophobic interactions, resulting in a stronger gel structure. Sensory evaluation suggested that surimi gels with psyllium husk powder were perceived as better than those without psyllium husk powder. The study concludes that selecting the appropriate psyllium husk powder quantity and thermal processing conditions based on salt concentration can significantly improve the quality of low-salt surimi gels. Error analysis using one-way ANOVA was performed on all experimental data and (*p* < 0.05) indicated the significant difference.

## 1. Introduction

Surimi is a mixture of salt-soluble proteins with gel-forming characteristics, obtained by eliminating unwanted elements such as lipids, pigments, blood, odorous compounds, and sarcoplasmic and myogenic proteins from fish meat [1]. Surimi products have gained global acceptance recently because of their appealing taste, texture, and nutritional content [2]. According to the China Fishery Statistical Yearbook report for 2023, China is known as one of the world’s largest fish producers. It produced 18.4 million metric tons (MMT) of silver carp (*Hypophthalmichthys molitrix*) [3]. Despite its high output and low price, silver carp faces issues such as fine thorns and an earthy smell in its meat [4]. Processing silver carp into surimi-based products, such as fish balls, kamaboko, fish sausages, tofu, and simulated crab meat, offers a valuable alternative [5,6].

Typically, surimi products were produced by adding salt (2–3%, *w*/*w*) [7]. However, in recent years, consumers are becoming aware of the health risks associated with a high-salt diet, such as the initiation of chronic diseases such as cardiovascular disease, osteoporosis, and hypertension. In this regard, the recommended daily intake (RDA) of salt is (2–5 g) according to the World Health Organization [8]. Currently, research related to surimi products is working to reduce the amount of salt in order to meet the consumers’ control of salt intake in their daily diet. However, the use of salt is crucial in the preparation of surimi products, and a reduction in the amount of salt added would inevitably be accompanied by a decrease in the quality of surimi products [9]. In this regard, researchers have explored a variety of techniques to improve the quality of low-salt surimi products, including the substitution of different salts, the use of new processing technologies, etc., but there is still room for further investigation [10,11,12,13]

Plantain belongs to the genus Plantago, family Plantaginaceae, and currently grows mainly in India, Pakistan, Iran, and some European countries [14]. Psyllium husk powder (PHP) is obtained by the ultrafine grinding technique and sieving of the outermost layer of the seed’s skin and is often used as a herbal medicine [15,16]. PHP is gaining attention among scientists for enhancing surimi gel properties due to its low-calorie content and beneficial attributes like hydrophilicity and colloidal properties [9,17]. Comprising 80% soluble and insoluble dietary fibers, PHP not only improves surimi gel properties but also aids digestion, reduces gastrointestinal issues, and lowers blood cholesterol, making it valuable in the pharmaceutical and food industries [18]. Recent studies have shown that PHP has positive effects on various food products like milk [19], yogurt [20], and meat patties [21], enhancing water retention and viscosity. Even a tiny addition of 0.1% PHP increased silver carp surimi gel strength [22]. Furthermore, in threadfin bream surimi, adding 1% PHP to 2.5% salt improved surimi products compared to those without PHP [23]. However, there is limited information on the impact of PHP at low salt concentrations.

Thermal conditions play a crucial role in the properties of surimi gels, involving three key steps, suwari, modori, and kamaboko, in the gelatinization process [24]. In addition, the use of other additives can complicate the process, such as the phase separation behavior of protein–polysaccharide complex systems during heat treatment [25,26]. Disulfide and non-disulfide covalent bonds were considered to be important forces in maintaining the mixed gel system of dietary fibers and myofibrillar proteins, and changes in ionic and hydrogen bonding were also affected by dietary fibers (*Lentinus edodes* stipes) [27]. And the formation or destruction of many intermolecular forces can be affected by the temperature and time of heat treatment [28]. Therefore, while exploring the effect of polysaccharide additions such as PHP on the gel quality of low-salt surimi, it is crucial to develop a matching thermotropic gelation procedure.

Our previous study explored the effects of PHP on silver carp surimi with the addition of 2.5% salt [22]. This study further investigated the impact of PHP amounts, heating times, and temperatures on the physicochemical characteristics of silver carp surimi at low salt levels (0.5% and 1%, *w*/*w*) considering our aim to reduce salt content. The aim of this study was to optimize process conditions of surimi gelation in low salt content while considering the effect of high salt consumption problems on human health and addition of PHP as a novel raw material due to high-fiber content making surimi more health-oriented. Our findings showed optimized surimi production with a low salt content, offering insights into the industry’s nutritious, healthy, high-quality surimi production.

## 2. Results and Discussions

### 2.1. Single-Factor Test

#### 2.1.1. Effect of Three Factors on Gel Strength of Surimi Gels

**Figure 1:**
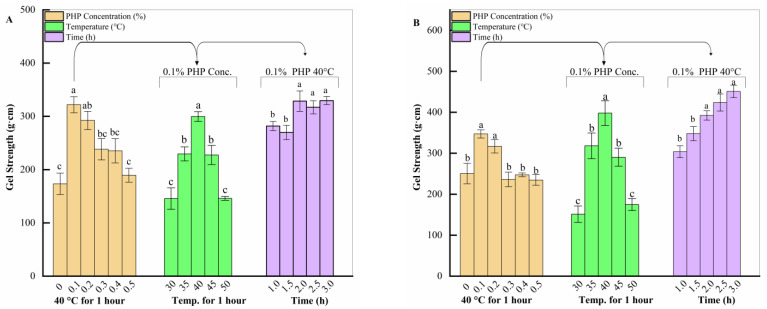
Effect of three factors on gel strength of surimi gels with varying salt contents ((**A**)—0.5%; (**B**)—1.0%, *w*/*w*). Different letters indicate significant differences between levels (*p* < 0.05).

The effects of three factors on the 0.5% (*w*/*w*) salt batch are presented in Figure 1A. The concentration-dependent samples exhibited the maximum gel strength at 0.1% (*w*/*w*) PHP, recorded as 321.85 ± 15.77 g.cm, significantly different from all samples (*p* < 0.05) except 0.2% (*w*/*w*) PHP samples (*p* > 0.05). Under different low-temperature treatment conditions, the treatment environment of 40 °C resulted in significant gel strength of surimi gel 298.56 ± 9.06 g.cm (*p* < 0.05). The time-focused samples had the highest gel strength of 348.83 ± 8.06 g.cm when incubated for 3 h at 40 °C. The results were significantly different from those of 1 h and 1.5 h treated samples (*p* < 0.05). However, the gel strength did not show any notable differences with 2 h and 2.5 h treatments (*p* > 0.05).

The effects of three factors on the 1% (*w*/*w*) salt group are shown in Figure 1B. The concentration-dependent samples demonstrated noticeable gel strength at 0.1% (*w*/*w*) PHP, recording 347.12 ± 9.73 g.cm, and were considerably different (*p* < 0.05) from all other samples except for the 0.2% (*w*/*w*) PHP sample (*p* > 0.05). The temperature-dependent samples exhibited higher gel strength at 40 °C, which was 396.37 ± 30.34 g.cm (*p* < 0.05). The time-focused treatments had the highest gel strength of 477.51 ± 16.41 g.cm when incubated for 3 h at 40 °C. The results were noticeably different with the 1 h and 1.5 h specimens (*p* < 0.05) but did not show significant difference after 2 h of heating.

Salt aids in solubilizing myofibrillar proteins within fish muscle, facilitating their extraction for gel formation [29]. Increasing salt content resulted in better gel strength in the same amount of PHP. Generally, 1% (*w*/*w*) salt samples displayed better gel strength, attributed to the extraction of more myofibrillar proteins and the resulting complex network. This observation aligns with previous studies [30,31,32]. A recent study identified the sequence of factors affecting gel strength as salt > time > heating method, which is consistent with our findings [33].

The 0.5% and 1% (*w*/*w*) salt content samples exhibited similar trends in three factors. Better gel strength was achieved with the addition of 0.1% and 0.2% (*w*/*w*) PHP. Adding an appropriate amount of PHP improved the surimi gel’s power, and the gel gradually changed from soft to complex elasticity. This could be attributed to PHP’s dietary fiber content. Its incorporation into the gel network can promote protein interactions with proteins and with water, resulting in a denser gel structure [17]. Additionally, dietary fiber’s strong water absorption allows it to compete for water within the gel network. An appropriate quantity of added PHP (<0.3%, *w*/*w*) helps to create a tight, three-dimensional gel network that retains moisture. However, excessive PHP (>0.3%, *w*/*w*) disrupts the gel network’s formation and stability, negatively impacting gel strength [13]. These findings are similar to those of Singh et al. [23], who reported that adding 1% (*w*/*w*) PHP to threadfin bream surimi (*Nemipteridae*) increased gel properties. Another study demonstrated that 2% yeast β-glucan in silver carp surimi improved textural properties. Still, higher concentrations reduced breaking force and breaking distance, decreasing gel strength [34]. Furthermore, addition of 15% (*w*/*w*) of soy protein and 8% (*w*/*w*) potato starch improved the gel strength of golden threadfin bream (*Nemipterus virgatus*) sausages. However, a further increase in concentration led to a decrease in gel strength [35].

Both salt-containing samples at varying temperatures exhibited similar trends regardless of salt content. As the temperature increases, the polysaccharide polymer chains’ motion increases, which is favorable to forming a denser protein gel system. Higher temperatures generally elevate the rate of protein solubility in surimi, thereby improving gel formation and network homogeneity by disrupting protein–protein interactions [36]. However, at 50 °C, enhanced endogenous protease activity in surimi can destroy the gel network, reducing gel strength [37].

Surimi gelation is time-dependent, with kinetics determining the rate of these processes. Over time, denatured protein molecules in surimi interact, forming protein–protein bonds like disulfide bonds, hydrogen bonding, and hydrophobic interactions [38]. These bonds contribute to cohesive gel network formation. As time passes, surimi gel undergoes structural changes, and gel strength gradually increases [39]. Protein rearrangement and cross-linking further strengthen the gel network, consistent with our results, regardless of salt content.

#### 2.1.2. Effect of Three Factors on Water-Holding Capacity of Surimi Gels

**Figure 2:**
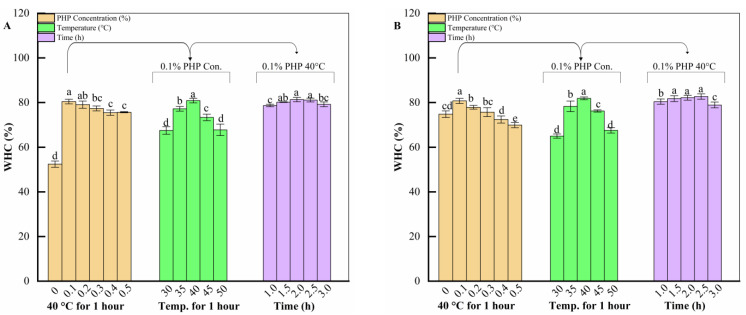
Effect of three factors on WHC of surimi gels with different salt contents ((**A**)—0.5%; (**B**)—1.0%, *w*/*w*). Different letters indicate significant differences between levels (*p* < 0.05).

Figure 2A shows the WHC of surimi gels of different one-factor treatment groups in the case of 0.5% (*w*/*w*) salt addition. Among the different additions, 0.1% (*w*/*w*) of PHP significantly enhanced the WHC of surimi gel to 80.46 ± 1.03% (*p* < 0.05), whereas, during the low-temperature treatment phase, treatment conditions at 40 °C were more favorable for the WHC of surimi gels (*p* < 0.05). The time-focused processing sample heated at 40 °C for 2 h showed a maximum WHC of 81.34 ± 0.99% and was markedly different (*p* < 0.05) than the 1 h and 3 h treated samples.

Figure 2B shows the WHC of surimi gels of different one-factor treatment groups in the case of 1.0% (*w*/*w*) salt addition. At this point, 0.1% PHP had an equally positive effect on the WHC of the surimi gel (*p* < 0.05). In addition, the low-temperature treatment environment of 40 °C and the low-temperature treatment time of 2.5 h were able to notably enhance the WHC of surimi gels in their respective one-way tests, reaching 81.866 ± 0.621% (*p* < 0.05) and 82.68 ± 1.20% (*p* < 0.05), respectively.

WHC measures a surimi protein gel’s ability to retain moisture and is a crucial characteristic of surimi products. Both salt 0.5% and 1% (*w*/*w*) content samples exhibited similar trends. As PHP was introduced, WHC initially increased and then decreased with further addition. Our results are consistent with those of Zhou et al. [17]. Moreover, these findings align with the study of Zhang et al. [40], suggesting that the incorporation of dietary fiber into the protein network weakens its water retention ability. The increased PHP content may reduce the relative protein content in surimi, leading to PHP insertion into the protein network, disrupting its structure and weakening water retention [34,41]. Similar trends were identified when farmed Meagre (*Argyrosomus regius*) muscle and silver carp surimi samples were added to PHP [22,30].

The WHC of 0.5% and 1% (*w*/*w*) salt content samples treated with 0.1% (*w*/*w*) PHP at different temperatures followed similar trends, consistent with the gel strength results. The gel system can generally be viewed as a network structure that traps water molecules [24]. Cellulose in dietary fiber contains β-1,4-glycosidic linkages, providing a wider surface area and strong cohesion for effective water absorption [42]. Elevated temperatures cause protein denaturation, involving structural unfolding and modification. Denaturation exposes hydrophilic regions in proteins, promoting more significant interaction with water molecules [36]. This enhanced interaction improves surimi’s water-holding capacity. On the other hand, excessively high temperatures (45 °C and 50 °C) may lead to deterioration of the surimi gel, allowing water to escape and reducing the WHC [43].

The trends in time-focused samples were also similar. However, the 0.5% (*w*/*w*) salt sample started releasing water earlier when set for over 2 h, unlike the 1% (*w*/*w*) salt sample, which began to remove water after 2.5 h at 40 °C. This difference may be attributed to their salt content. The lower salt content sample may struggle to retain moisture for an extended period compared to the higher salt content sample, which forms a more complex matrix structure. Gao et al. [33], who studied the effect of different salt contents and times in HIU-treated silver carp surimi, found that salt significantly impacted gel properties and WHC. Our results are consistent with theirs, as the 1% (*w*/*w*) salt sample exhibited higher WHC than the 0.5% (*w*/*w*) salt sample.

### 2.2. Orthogonal Test Result Analysis

The specific test plan and results analysis are shown in Table 1 for the 0.5% (*w*/*w*) salt batch. Based on the single-factor test results, for the gelation temperature, gelation time, and PHP addition, using the three-factor three-level orthogonal table L9 (34), the gel strength and WHC (both weighted at 50%) were tested for the evaluation system (Section 4.5). From the intuitive research in Table 1, it can be found that the optimal solution for surimi gel is A_1_B_3_C_3,_ and the combined score is 9.85 points; the concentration, temperature, and time of PHP evaluated were 0.3% (*w*/*w*) PHP at 35 °C for 3 h. Intuitive analysis results for A_1_B_3_C_3_ differed from computational analysis results for A_2_B_3_C_2_, so a verification test was required.

Analysis of the ideal combination within 1% (*w*/*w*) salt-containing samples yields the outcomes presented in Table 2. An intuitive analysis of the table indicates that A_1_B_2_C_2_ emerges as the optimal solution for surimi gel with combined scores of 10.00 points. The associated conditions include a temperature of 35 °C, a gelation time of 2.5 h, and the addition of 0.2% (*w*/*w*) PHP. Notably, the visual analysis results for A_1_B_2_C_2_ in 1% (*w*/*w*) salt samples diverged from the computational analysis A_1_B_1_C_3_, prompting the need for a verification test.

### 2.3. Verification Test

Following a verification test that integrated intuitive and computational analyses for the 0.5% (*w*/*w*) salt batch (Table 3), it became apparent that the combined score for A_2_B_3_C_2_ was lower than that of A_1_B_3_C_3_. Subsequent verification tests confirmed that the No. 11 combination plan, A_1_B_3_C_3_, retained its status as the optimal choice, aligning with the findings from the first batch scheme No. 3 combination plan (Table 1). Consequently, the definitive solution for surimi gel was identified as A_1_B_3_C_3_, featuring a gelation temperature of 35 °C, a gelation time of 3 h, and adding PHP at a concentration of 0.3% (*w*/*w*).

Our findings revealed that the combined score of A_1_B_2_C_2_ in the verification test aligned with that obtained in the orthogonal experiment for 1% (*w*/*w*) salt samples (Table 2). Subsequently, the second round of verification tests (Table 3) confirmed that the No. 13 combination plan, A_1_B_2_C_2_, exhibited similar performance to the initial batch combination scheme, A_1_B_2_C_2_ (Table 2). Consequently, the ultimate determination for the optimal surimi gel solution settled on A_1_B_2_C_2_, specifying a gelation temperature of 35 °C, a gelation time of 2.5 h, and the addition of PHP at a concentration of 0.2% (*w*/*w*).

### 2.4. Gel Strength and WHC

**Figure 3:**
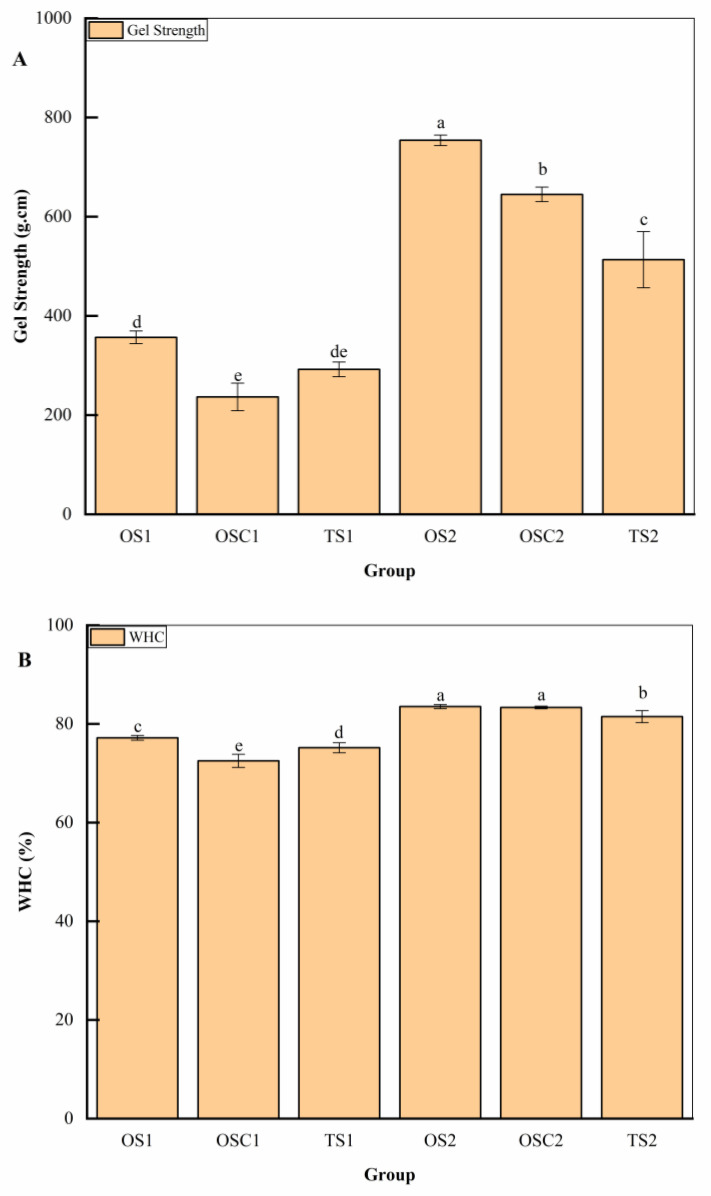
Gel strength (**A**) and WHC (**B**) of surimi gels of each validation group. Different letters indicate significant differences between levels (*p* < 0.05).

Figure 3A shows that the addition of PHP noticeably (*p* < 0.05) impacted gel strength in the optimized group. This implies that the addition of PHP strengthened the gel structure, ultimately increasing the gel strength of the optimized group. The lower gel strength observed in the OSC_1_ sample may be attributed to an extended setting time in low salt content. This prolonged time might have disrupted the network structure due to reduced protein solubility in low salt content, resulting in lower gel strength for the sample. In contrast, in 1% salt samples, higher protein solubility helped maintain the gel network even for a longer duration without the presence of PHP. Similar results were reported by Zhu et al. [22].

The results of WHC are consistent with the gel strength, as indicated in Figure 3B. The addition of PHP also significantly impacted WHC. WHC measures water availability throughout the dense gel network system. The OS_1_ and OS_2_ samples showed notable (*p* < 0.05) WHC compared to their corresponding samples. OSC_1_ treatments showed the lowest WHC, consistent with gel strength results. The WHC of OSC_2_ and OS_2_ showed no significant difference (*p* > 0.05). This lack of difference might be attributed to the lower amount of PHP added to OS_2_ compared to OS_1_. However, it did not significantly improve WHC compared to OSC_2_. Nevertheless, both OS_2_ and OSC_2_ demonstrated significantly higher WHC than TS_2_, indicating that not only did PHP enhance WHC, but the higher salt content and longer setting time also impacted the WHC of surimi gel. The addition of tapioca starch increased the WHC of (*Virgatus nemipterus*) surimi gel, with a higher amount resulting in higher WHC [44]. This outcome is also in line with the analysis of Cardoso and Mendes [30], who observed that the WHC of Meagre (*Argyrosomus regius*) muscle gel also improved when incorporated with psyllium dietary fiber.

### 2.5. Textural Profile Analysis

TPA is often used to evaluate food products’ mechanical qualities. The OS_1_ sample showed more hardness than OSC_1_ and TS_1_. OS_1_ sample had a significant difference in hardness from the TS_1_ sample (*p* < 0.05), but as compared to the OSC_1_ sample, there were no noticeable differences between them (*p* > 0.05) as shown in Table 4. The highest hardness values were demonstrated by OS_2_ 9784.9173 ± 151.7795 and were remarkably different from those of TS_2_. However, OS_2_ and OSC_2_ group hardness results were not significantly different (*p* > 0.05).

In the OS_1_ sample, the PHP concentration (0.3%, *w*/*w*) was higher as compared to OS_2_ (0.2%, *w*/*w*), and heating time (3 h) was also higher. Hence, higher hardness and chewiness were observed compared to OSC_1_ and TS_1,_ whereas OS_1_ and OSC_1_ had no significant differences in values, suggesting that not only did PHP affect the gel, but also the optimal temperature and time improved the gel quality.

Meanwhile, in OS_2_ samples, PHP concentration had less effect on surimi gumminess, cohesiveness, chewiness, and resilience, while OSC_2_ samples showed higher values. Hence, it can be speculated here that PHP has a more significant effect on surimi hardness. Overall, the optimal temperature and time were able to improve the texture characteristics of surimi products to some extent compared to the traditional group. Moreover, elevated salt levels promote higher hardness in gels [45]. Moreover, our results are consistent with the study reported by Huang et al. [44] as the presence of tapioca starch increased the hardness and chewiness of *Virgatus nemipterus* surimi gel. Furthermore, another study conducted by Shahsavani Mojarrad et al. [46] reported enhancement in hardness values when amylose corn starch was added to wheat starch composite gels.

Adhesiveness is the force required to overcome the forces between the surimi sample and the probe (or tongue, palate, or teeth) [47]. Minimum adhesiveness was recorded in the optimized group. Cohesiveness can be determined by the amount of compression between two surfaces before they break by dividing the area under the force–time curve between the first and second compression cycles. Samples with PHP had shown lower values for cohesiveness.

Chewiness reflects the energy needed to chew food before swallowing, which is positively correlated with hardness [48]. Both hardness and chewiness are linked to fish protein quality [49]. A previous study found that curdlan improved textural properties in low-fat and low-salt sausage and increased hardness at 1.5% (*w*/*w*) salt [50]. However, this suggests that the presence of PHP leads to a firmer gel structure, requiring more energy for chewing solid food.

### 2.6. Intermolecular Forces

**Figure 4:**
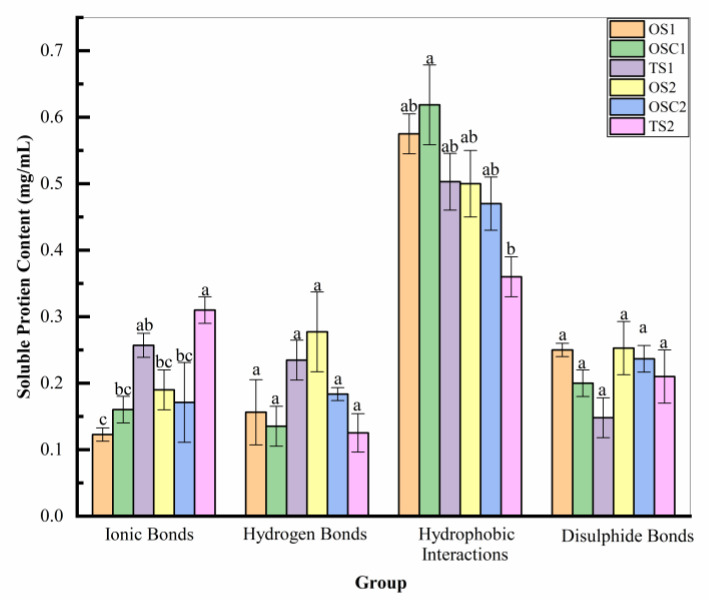
Chemical forces between surimi samples under different process conditions: Different letters indicate their significant difference (*p* < 0.05) between same bonding groups. OS_1_, OS_2_ (optimized surimi), OSC_1_, OSC_2_ (optimized surimi control), TS_1_, TS_2_ (traditional surimi). Samples with digit 1 have 0.5% (*w*/*w*) salt and with digit 2 have 1% (*w*/*w*) salt.

The bonds of hydrogen, ionic, disulfide, and hydrophobic interactions of the soluble protein in the surimi gels in the different treatments are shown in Figure 4. According to these results, the ionic bond content of the optimized group was significantly reduced (*p* < 0.05) compared to traditional group in both 0.5% and 1% (*w*/*w*) salt batches. Higher hydrogen bonding content was exhibited in OS_2_ and OSC_2_ compared to OS_1_ and OSC_1_. No significant changes in hydrogen bonding content were observed in the treatment groups. Hydrophobic interactions in OSC_1_ had a high content. The disulfide bond, which is an important force in maintaining the gel structure of surimi, did not show significant changes in the content in any of the treatment groups. The batch with 0.5% (*w*/*w*) salt had higher hydrophobic interactions than the 1% (*w*/*w*) salt batch. At the same time, ionic and disulfide bonds seemed higher in 1% (*w*/*w*) salt samples.

The decrease in ionic bonds in the optimized group could be due to the elevated setting time of surimi gels compared to traditional group. More gelation time promotes more protein denaturation, increasing cross-linking between the proteins. According to Liu et al. [51], setting time resulted in decreased ionic bonds and increased hydrophobic interactions. The ionic bonds were higher in TS_2_ than TS_1_, likely due to the abundance of more ions in the water system than in the low salt.

Optimized samples with PHP showed more excellent hydrogen bonding than samples without PHP, and, being hydrophilic, they can contribute additional polar functional groups. These groups can potentially form hydrogen bonds with water molecules and with other polar groups in the proteins of surimi [52]. At the same time, hydrophobic interactions in TS_1_ were higher compared to TS_2_. This could be due to the high concentration of salt in TS_2_ samples, which may promote high cross-linking in high salt content as there would be more ions to compete for water molecules that could raise the hydrophobic TS_1_ interactions and reduce TS_2_ into other bonds like ionic bonds or hydrogen bonds. The OS_2_ and OSC_2_ samples also showed fewer hydrophobic interactions than OS_1_ and OSC_1_.

Moreover, the creation of the three-dimensional network structure of surimi gels can be markedly accelerated by aggregating the hydrophobic groups of the connected proteins [51]. Hydrophobic interactions are the primary chemical interactions responsible for stabilizing the surimi gel network, but ionic and hydrogen bonds also play a minor role [53]. Prolonged heating can expose myosin hydrophobic groups and cause denaturation of proteins. This is advantageous for the aggregation and cross-linking of proteins with PHP [54]. According to the report, the textural properties of silver carp surimi gels can be enhanced by the “filling effect” and “water absorption (dehydration) effect” of nano-sized Okara insoluble dietary fibers [55]. Similar effects might be attributed to the dietary fibers in PHP, which could help myofibrillar proteins (MPs) create hydrophobic connections.

The optimized group might cause the change in protein structure by uncovering more sulfhydryl groups for a longer time, which would assist the oxidization in forming disulfide bonds, enhancing the cross-linking among proteins, and resulting in a more uniform network structure. Due to the low setting temperature in the optimized group, it cannot be significantly different from the traditional group [56].

### 2.7. Composition of Proteins

**Figure 5:**
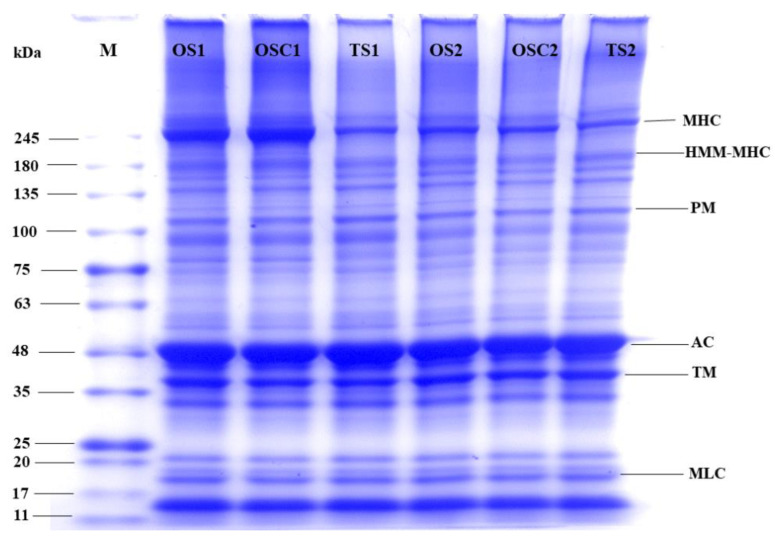
Protein composition in surimi gels with different salt and process conditions. OS_1_, OS_2_ (optimized surimi), OSC_1_, OSC_2_ (optimized surimi control), TS_1_, TS_2_ (traditional surimi). Samples with digit 1 have 0.5% (*w*/*w*) salt and with digit 2 have 1% (*w*/*w*) salt.

SDS PAGE results are shown in Figure 5, displaying that the myosin-heavy chain (MHC) bands of OS_2_ and OSC_2_ have lighter bands than OS_1_ and OSC_1_. The study found that the surimi paste contained the main MHC and actin (AC) proteins [57]. This indicates that higher salt content caused more MHC aggregation because the more MHC aggregation there was, the more difficult it was to enter the electrophoresis gel [58]. During the gelation of surimi paste, MHC molecules combine to create complex aggregates, eventually forming a network structure, so MHC bands gradually disappear in OS_2_ and OSC_2_. Hence, this indicates that OS_2_ and OSC_2_ are more conducive to developing surimi gel. On the other hand, TS_2_ has no significant difference from TS_1_, which is enough to show that optimization can enhance the gel properties of low-salt surimi.

However, compared to MHC, AC was the dominating protein in the gel, showing that actin has a high resilience to protein degradation or has a lower role in polymerization during the gelation process [59]. Moreover, the tropomyosin (TM) and myosin light chain (MLC) are the lower-molecular-weight proteins. Furthermore, they indicated no significant results in both optimized and control samples exhibited minimal changes among the three process conditions, suggesting that these proteins may not play a substantial role in gel formation [60].

### 2.8. Microstructure

**Figure 6:**
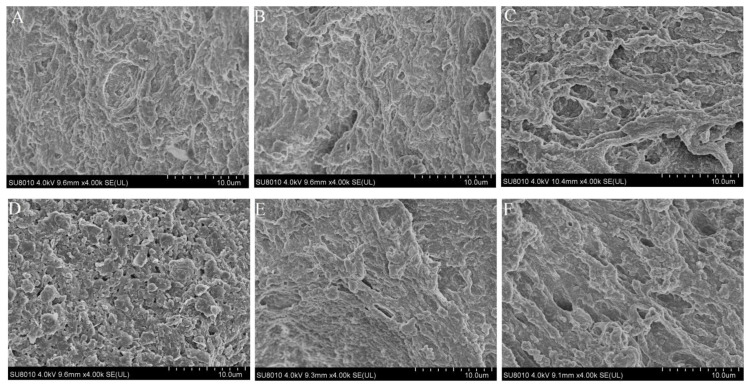
Microstructure of surimi samples with a magnification of (×4000). (**A**) OS_1_, (**B**) OSC_1_, (**C**) TS_1_, (**D**) OS_2_, (**E**) OSC_2_, and (**F**) TS_2_ groups.

The SEM technique was employed to study the microstructure of silver carp surimi gel. Results indicated that the salt content and PHP in 0.5% and 1% (*w*/*w*) salt groups significantly influence the silver carp surimi gel structure, as shown in Figure 6. Surimi samples with PHP exhibited denser, more uniform, and compact gel structures than those without PHP. Samples without PHP had more porous gel structures, with increasing salt content leading to reduced porosity and enhanced surface uniformity [61].

Heated myofibrillar proteins in surimi undergo denaturation and aggregation, forming a dense three-dimensional network structure. Traditional two-stage water bath processing results in a grainy surimi gel surface, while the OSC_1_ and OSC_2_ gel surfaces remain rough and uneven compared to the smoother optimized samples. The increased cross-linking suggests that PHP may fill gaps between protein molecules, enhancing the gel matrix density. This aligns with previous findings for silver carp surimi treated with PHP [22]. Combining MPs with 0.5% (*w*/*w*) PHP resulted in a dense microstructure network, as demonstrated by Zhou et al. [17], indicating PHP’s positive impact on surimi samples in both salt levels.

### 2.9. Sensory Evaluation

Table 5 shows the result of sensory evaluation for the optimized and traditional groups under three process conditions. The optimized surimi (OS_2_) showed significant difference (*p* < 0.05) compared to its corresponding samples in consumer acceptability. Overall, the sample with 1% salt content achieved higher scores for all attributes. The most liked specimen was OS_2_, whereas OSC_1_ was perceived as less liked sample. All sensory scores were more than 4, which means no samples were disliked by the consumers and had no adverse effect on consumer acceptability. In initial appearance, the samples with 1% salt content scored higher and were significantly different from OSC_1_ and TS_1_ (*p* < 0.05). Moreover, they scored slightly higher compared to 0.5% salt samples in flavor properties. However, the optimized surimi control (OSC_1_) with 0.5% salt scored lowest 4.95 ± 0.94^b^ among all other samples. This might be due to the prolonged heating in the presence of low salt, which might have suppressed its flavor and disrupted its texture. Furthermore, the optimized sample (OS_2_) achieved the highest scores in flavor, significantly different from OSC_1_. This might be due to perceived saltiness with the combination of a minute amount of PHP enhancing its flavor properties. The panelists were unable to differentiate between the aroma profile of all the samples significantly (*p* > 0.05). Singh et al. [23] reported similar results when samples were added to threadfin bream surimi. This might be due to the fact that PHP does not have a strong flavor or odor, which means its inclusion at low levels is unlikely to impact the sensory characteristics of foods significantly, while enhancing functional properties could be considered a successful outcome. Overall, panelists preferred optimized surimi (OS_2_), whereas OSC_2_ was less liked, and there were significant differences (*p* < 0.05) in the outcomes of every attribute of samples added with PHP to control samples. Only the perceived saltiness made them differentiate between the samples slightly. Textural properties of OSC_1_ were perceived to be inferior to other samples. Overall higher scores were also obtained by the samples with higher content of salt, and panelists reported no significant difference in the samples with or without PHP.

## 3. Conclusions

This research explored how PHP addition, gelation temperature, and gelation time influence surimi gel’s gel strength and water-holding capacity (WHC) for two salt concentrations (0.5% and 1%, *w/w*). Through orthogonal experimentation, it was found that optimal conditions were achieved with 0.3% (*w/w*) PHP for 0.5% (*w/w*) salt and 0.2% (*w/w*) PHP for 1% (*w/w*) salt at a temperature of 35 °C for durations of 3 and 2.5 h, respectively. The study also observed, via SDS-PAGE analysis, that PHP likely promotes the cross-linking of MHC molecules. SEM analysis showed that PHP contributes to a smoother and denser texture in the OS_1_ and OS_2_ groups compared to OSC_1_ and OSC_2_ and the TS_1_ and TS_2_ groups, with OSC_1_ and OSC_2_ exhibiting superior structure compared to the TS groups. This suggests that the ideal PHP concentration, temperature, and time can enhance surimi’s quality. Consistent with these findings, the texture profile analysis (TPA) demonstrated that PHP addition increased gel hardness in both salt conditions. Furthermore, PHP’s incorporation resulted in reduced ionic bonds and marginal enhancements in hydrophobic interactions and disulfide bonds. Consumer acceptance tests indicated that the PHP-optimized samples were well-received and preferred by the consumer. Future studies should delve into the interactions of PHP with surimi proteins, its effects on water distribution, and its influence on the surimi gel’s secondary structure.

The current study optimized the silver carp surimi gel in the presence of low salt considering health problems. In future research, comparison of the nutritional profile of optimized surimi compared to the traditional method can be conducted. Moreover, other hydrocolloids and amino acids have the potential to enhance surimi gel properties and can be used individually or in their combinations to assess their effects in optimizing silver carp surimi.

## 4. Materials and Methods

### 4.1. Materials

AAA-mark frozen silver carp surimi was provided by Honghu Jingli Aquatic Food Co., Ltd., Jingzhou, China, and kept at −18 °C for no more than 2 months. PHP (99% purity, *w*/*w*) was purchased from Shanghai Lexiang Biotechnology Co., Ltd., Shanghai, China. The food-grade plastic casings and NaCl were bought from neighboring supermarkets. Solarbio Science & Technology Co., Ltd. (Beijing, China) supplied all of the chemicals utilized, which were of analytical quality.

### 4.2. Single-Factor Test

#### Preparation of Surimi Gels with Different PHP Additions, Temperature, and Time

The thawed surimi was chopped using a chopper, and salt (0.5% and 1%, *w/w*) as well as different concentrations of PHP (0%, 0.1%, 0.2%, 0.3%, 0.4%, and 0.5%) were added to mix with the surimi, and moisture content was maintained at 78% by using moisture analyzer. The mixture was extruded into a polyvinyl chloride plastic casing and heated in a water bath and incubated at 40 °C for 60 min and then at 90 °C for 30 min.

To analyze surimi gel behavior at different temperatures based on gel strength and WHC results, 0.1% PHP was added to salt (0.5% and 1% *w/w*), mixed with the surimi, and incubated at low temperatures (30 °C, 35 °C, 40 °C, 45 °C, and 50 °C) for 60 min and then at 90 °C for 30 min.

For the analysis of the effect of different time durations on surimi gel based on gel strength and WHC results, 0.1% PHP was added to salt (0.5% and 1% *w/w*) and mixed with surimi. The mixture was then heated at 40 °C for different times (1.0, 1.5, 2.0, 2.5, and 3.0 h) and then at 90 °C for 30 min. All samples were cooled down in running water and stored at 4 °C.

### 4.3. Determination of Gel Strength

The previously made surimi gels were sliced into 2 cm long cylinders after being left at room temperature for approximately one hour. A TA-XT texture analyzer (Stable Micro System) was used to determine the breaking force (g) and deformation (cm). We followed the settings and parameters outlined by Shahsavani Mojarrad et al. [62] with minor adjustments: penetration mode, P/5S spherical probe (diameter = 5 mm), velocity = 1.0 mm/s, pre-test velocity = 1.0 mm/s, post-test velocity = 10 mm/s, test recovery velocity = 10.0 mm/s, target mode = distance, pressing distance = 30 mm, trigger type = auto (force), and trigger force = 5.0 g. Gel strength was computed using the provided formula, and tests were conducted in septuplicate.

(1) Breaking force (g)×Deformation (cm)=Gel strength (g·cm)

### 4.4. Water-Holding Capacity (WHC)

The analysis of WHC by using a UK CT15RT desktop high-speed refrigerated centrifuge was conducted based on the technique suggested by Liang et al. [63] with minor variations. Gel fragments of 2 mm thickness (2–3 g) were precisely balanced (W_1_) and infolded in three films of filter paper. Each sample was filed in a 50 mL centrifuge tube at (25 °C), centrifugated at 8000× *g* for 10 min, and then balanced again (W_2_) without filter paper. The WHC of the sample was computed using the following formula, and parallel tests were conducted in triplicate.

(2) $$\mathrm{WHC}\;\left(\%\right)=\frac{W_{2}}{W_{1}}\text{×}100$$

### 4.5. Orthogonal Test

The surimi gel preparation process took WHC and gel strength as the evaluation criteria and selected gelation temperature (A), gelation time (B), and PHP addition amount (C) according to the single factor test results. An orthogonal experimental design, denoted as L_9_ (3^4^), was implemented across three levels of three factors. The factor level table is shown in Table 6.

To calculate the degree of gel strength (*Yi*_1_) and WHC (*Yi*_2_), the following formula was used (Table 1, Table 2 and Table 3).

(3) $${Yi}_{1}=\frac{{yi}_{1}-yi_{min}}{y1_{max}-y1_{min}}\;,\;{Yi}_{2}=\frac{yi_{2}-yi_{min}}{y2_{max}-y2_{min}}$$

*yi*_1_ is the actual values of gel strength, and *yi*_2_ is the actual values of WHC obtained during the experiment mentioned in the orthogonal experiment column.

Following the equation, the combined scores of samples were obtained as

(4) $$Yi=\sum_{j=1}^{2}B_{j}Yi_{j}=B_{1}Yi_{1}+B_{2}Yi_{2}$$

*Yi* is the final combined scores of samples acquired by calculating *Yi*_1_ and *Yi*_2_.

Whereas,

(5) $$Yi=B_{1}Yi_{1}+B_{2}Yi_{2}=0.5\times Yi_{1}+0.5\times Yi_{2}$$

The *B*_1_ and *B*_2_ are the weight coefficients of gel strength and WHC.

Gel strength and WHC are equally weighted.

### 4.6. Validation and Comparison of Optimized Parameters

#### 4.6.1. Preparation of Surimi Gel

For the 0.5% (*w*/*w*) salt batch, traditional surimi (TS_1_) was prepared with the primary phase at 40 °C for 1 h and the next at 90 °C for 30 min without PHP. The optimized surimi (OS_1_) followed the optimal plan with the first heating at 35 °C for 3 h, the second water bath heating at 90 °C for 30 min, and the addition of 0.3% (*w*/*w*) PHP. An optimized surimi control (OSC_1_) used the same conditions as OS_1_ but without PHP.

For the 1% (*w*/*w*) salt batch, traditional surimi (TS_2_) was prepared with the initial phase at 40 °C for 1 h and the following phase at 90 °C for 30 min without PHP. The optimized surimi (OS_2_) followed the ideal plan with the first water bath at 35 °C for 2.5 h, the following water bath at 90 °C for 30 min, and 0.2% (*w*/*w*) PHP added. An optimized surimi control (OSC_2_) used the same conditions as OS_2_ but without PHP. Samples including OS_1,_ OS_2_, OSC_1,_ and OSC_2_ are termed as the optimized group generally, whereas those containing TS_1_ and TS_2_ are designated as the traditional group.

#### 4.6.2. Textural Profile Analysis

The samples were examined using a TA-XT Plus texture analyzer and a 50 mm thick cylindrical aluminum probe (P/36R) according to the method of Shahsavani Mojarrad et al. [46] with minor adjustments. The test, pre-test, and post-test velocities were set at 1.0 mm·s^−1^, target mode = strain with 50% strain, time = 5.0 s, trigger type = auto (force), and tare mode = auto, and advanced options were set to ON mode. The hardness can be defined as a measure of maximum force at any time during the first compression cycle. Springiness as the ratio between recovered height after the first compression and the original gel height, adhesiveness as the negative area of the curve during retraction of the probe, and cohesiveness as the ratio of the work area during the second compression to the work area during the first compression were determined. The later determines the degree of difficulty in breaking down the internal gel structure. Gumminess is calculated as the product of hardness and cohesiveness, whereas chewiness is a parameter that describes the energy required to chew a solid food product to a state where it can be swallowed. In addition, resilience measures the ability of a food product to return to its original form after the removal of a deforming force. The textural profiles, including hardness, cohesiveness, springiness, gumminess, adhesiveness, resilience, and chewiness, were recorded in septuplicate.

#### 4.6.3. Chemical Interactions

Chemical forces were determined using the technique of Zhu et al. [22] with minor amendments. About 2 g of the ground gels were independently immersed in each of the resulting mixtures: (SA) 0.05 mol/L NaCl, (SB) 0.6 mol/L NaCl, (SC) 0.6 mol/L NaCl + 1.5 mol/L urea, and (SD) 0.6 mol/L NaCl + 8 mol/L urea and (SE) 0.6 mol/L NaCl + 8 mol/L urea + 0.5 mol/L β-mercaptoethanol. The blend was homogenized with a T25 Ultra-Turrax homogenizer (IKA Labortechnik, Staufen, Germany) for 3 min at 23,000 rpm and permitted to set at 4 °C for 1 h. The mixture was then centrifugated at 10,000 rpm for 15 min at 4 °C. The protein content was calculated by determining the supernatant using the Coomassie brilliant blue method in triplicate. The index of hydrogen and ionic bonds, hydrophobic interactions, and bonds of disulfide was stated by the variance in supernatant protein content using the following Equations.

Index of ionic bonds = SB − SA Index of hydrogen bonds = SC − SB Index of hydrophobic interactions = SD − SC Index of disulfide bonds = SE − SD(6)

#### 4.6.4. Sodium Dodecyl-Sulfate Polyacrylamide Gel Electrophoresis (SDS-PAGE)

Surimi gel SDS-PAGE followed the method of Zhu et al. [58] with minor adjustments. SDS solution (27.0 mL) was inserted into 3 g of the sample, homogenized at 12,000 rpm for 2 min, and set at 85 °C for 1 h. The supernatant was assessed after spinning at 10,000 rpm in a centrifuge for 15 min. Supernatant protein dilution was adjusted to 5 mg/mL and mixed 1:1 (V: V) with a loading buffer. The blend was heated for 5–7 min, and 5 µL of the sample was loaded onto a polyacrylamide gel (4% concentrate gel and 12% separating gel). Electrophoresis was operated at a constant current of 80 V for 90 min using a DYY-Type 11 electrophoresis apparatus from Shanghai Tianmei Biochemical Instrument and Equipment Engineering Co., Ltd. After the electrophoretic process, proteins were stained with Coomassie Brilliant Blue R-250 and decolorized in distilled water, and the gels were photographed and analyzed.

#### 4.6.5. Scanning Electron Microscopy (SEM)

The microscopic structures of the gels were examined using the approach previously suggested by Lu et al. [12] with slight variations. The samples were sliced into 2 mm thick rectangular fragments, immersed in a 2.5% glutaraldehyde mixture overnight to fix, and then washed with distilled water three times for 10 min. The resultant samples were subjected to rapid dehydration for 10 min using solutions containing 50%, 70%, 80%, 90%, and 100% (*v*/*v*) ethanol. After freeze-drying in a vacuum freeze drier (FD-1B- 50, Beijing Bo Medical Kang Experimental Instrument Co., Ltd., Beijing, China), ion sputter gold plating was applied. Then, the microstructure of the gel samples was analyzed at an accelerating voltage of 4.0 kV and magnifications of (4000×) using a Regulus SU8010 (Hitachi, Tokyo, Japan) scanning electron microscope.

#### 4.6.6. Sensory Evaluation

The acceptability test of the optimized group and traditional group with 0.5% and 1% salt was carried out with the 7-point hedonic scale as per the method of Wang et al. [40]. The scale was set as 1 (extremely dislike), 2 (moderately dislike), 3 (slightly dislike), 4 (neither like nor dislike), 5 (slightly like), 6 (moderately like), and 7 (extremely like). In this study, twenty food technologist panelists (10 males and 10 females) were recruited from the campus of Hefei University of Technology (Hefei, China). Samples were placed on the paper plates. The five attributes were appearance, aroma, flavor, texture, and overall scores of surimi gel. Each sample was coded with a randomly selected three-digit number. Panelists were instructed to use water to clean their palates between the samples. Surimi gels were considered acceptable if their mean overall scores were above 4 (neither like nor dislike). All samples were evaluated under the same conditions.

### 4.7. Statistical Analysis

According to the single-factor test results, the orthogonal test of L_9_(3^4^) was carried out with Design-Expert software 13, the water-holding ability and gel strength were used as the evaluation criteria, and the combined scoring method was adopted. Considering the significance of the two indicators, water-holding capacity, and gel strength, weight coefficients of 0.5 were assigned to each. The weighted sum was then calculated to derive the combined score, leading to the identification of the optimal process conditions. Error analysis using one-way ANOVA was performed on all experimental data, expressed as the mean ± standard deviation, and the significance test was executed by SPSS 20.0 software, and (*p* < 0.05) indicated a significant difference.

## Figures and Tables

**Table 1:** Results of orthogonal experiments with L_9_ (3^4^) for surimi gels under 0.5% (*w*/*w*) salt content conditions.

Serial No.	Temp. °C (A)	Time H (B)	Conc. % (C)	Gel Strength (g·cm) (yi_1_)	WHC (%) (yi_2_)	Gel Strength Degree (Yi_1_)	WHC Degree (Yi_2_)	Comb. Score (Yi)
1	1 _(35)_	1 _(2)_	1 _(0.1)_	213.20	57.04	0.00	0.00	0.00
2	1	2 _(2.5)_	2 _(0.2)_	273.23	68.68	0.59	0.70	6.45
3	1	3 _(3)_	3 _(0.3)_	311.65	73.64	0.97	1.00	9.85
4	2 _(40)_	1	2	304.88	67.56	0.91	0.63	7.70
5	2	2	3	264.20	69.79	0.50	0.76	6.30
6	2	3	1	313.66	68.81	1.00	0.70	8.50
7	3 _(45)_	1	3	226.76	64.50	0.13	0.40	2.65
8	3	2	1	237.39	65.54	0.20	0.50	3.50
9	3	3	2	281.70	62.16	0.68	0.30	4.90
K_1_	16.30	10.20	12.00					
K_2_	22.5	16.25	19.05					
K_3_	11.05	23.25	18.80					
Range	11.45	13.05	7.05					
Factors, major to minor	BAC							
Optimal conditions	A_2_B_3_C_2_							

**Table 2:** Results of orthogonal experiments with L_9_(3^4^) for surimi gels under 1.0% (*w*/*w*) salt content conditions.

Serial No.	Temp °C (A)	Time H (B)	Conc. % (C)	Gel Strength (g·cm) (yi_1_)	WHC (%) (yi_2_)	Gel Strength Degree (Yi_1_)	WHC Degree (Yi_2_)	Comb. Score (Yi)
1	1 _(35)_	1 _(2)_	1 _(0.1)_	415.25	77.81	0.82	0.81	8.15
2	1	2 _(2.5)_	2 _(0.2)_	449.05	80.92	1.00	1.00	10.0
3	1	3 _(3)_	3 _(0.3)_	436.79	80.85	0.93	0.99	9.60
4	2 _(40)_	1	2	419	80.53	0.84	0.97	9.05
5	2	2	3	428.67	78.78	0.89	0.87	8.80
6	2	3	1	361.04	76.58	0.55	0.73	6.40
7	3 _(45)_	1	3	273	78.18	0.10	0.83	4.65
8	3	2	1	252.56	64.35	0.00	0.00	0.00
9	3	3	2	260.49	69.66	0.04	0.32	1.80
K_1_	27.75	21.85	14.55					
K_2_	24.25	18.18	20.85					
K_3_	6.45	17.8	23.05					
Range	21.3	4.05	8.5					
Factors, major to minor	ACB							
Optimal conditions	A_1_B_1_C_3_							

**Table 3:** 0.5% and 1.0% (*w*/*w*) salt content-based surimi gel properties verification test.

Salt %	Serial No.	Temp °C (A)	Time H (B)	Conc. % (C)	Gel Strength (g·cm) (yi_1_)	WHC (%) (yi_2_)	Gel Strength Degree (Yi_1_)	WHC Degree (Yi_2_)	Comb. Score (Yi)
0.5	10	2 _(40)_	3 _(3)_	2 _(0.2)_	205.43	59.38	0.00	0.00	0.00
	11	1 _(35)_	3	3 _(0.3)_	282.80	61.48	1.00	1.00	10.0
1.0	12	1	1 _(2)_	3	395.17	73.57	0.00	0.00	0.00
	13	1	2 _(2.5)_	2	433.5	75.83	1.00	1.00	10.0

**Table 4:** Textural profile analysis of 0.5% and 1% (*w*/*w*) salt content samples.

Sample	Hardness (g)	Adhesiveness (g. sec)	Springiness (%)	Cohesiveness (%)	Gumminess (g)	Chewiness (g)	Resilience (%)
OS_1_	8723.86 ± 76.72 ^b^	−187.70 ± 101.42 ^a^	0.87 ± 0.00 ^bc^	0.68 ± 0.02 ^c^	5913.16 ± 212.90 ^c^	5133.96 ± 210.13 ^c^	0.36 ± 0.01 ^c^
OSC_1_	8567.35 ± 97.71 ^bc^	−159.67 ± 91.47 ^a^	0.86 ± 0.01 ^c^	0.70 ± 0.01 ^bc^	5988.61 ± 182.32 ^c^	5099.54 ± 121.23 ^c^	0.37 ± 0.01 ^c^
TS_1_	8114.14 ± 208.24 ^d^	−85.56 ± 71.65 ^a^	0.87 ± 0.01 ^b^	0.71 ± 0.01 ^b^	5763.86 ± 140.65 ^c^	5033.50 ± 134.20 ^c^	0.37 ± 0.37 ^c^
OS_2_	9784.92 ± 151.78 ^a^	−130.50 ± 109.94 ^a^	0.91 ± 0.02 ^a^	0.77 ± 0.03 ^a^	7564.82 ± 251.99 ^a^	6879.20 ± 172.43 ^a^	0.47 ± 0.00 ^a^
OSC_2_	9606.17 ± 110.64 ^a^	−149.13 ± 79.94 ^a^	0.91 ± 0.01 ^a^	0.79± 0.01 ^a^	7574.97 ± 181.92 ^a^	6906.84 ± 212.04 ^a^	0.48 ± 0.00 ^a^
TS_2_	8310.89 ± 327.94 ^cd^	−109.43 ± 104.51 ^a^	0.90 ± 0.02 ^a^	0.77 ± 0.01 ^a^	6414.06 ± 220.27 ^b^	5756.66 ± 183.40 ^b^	0.45 ± 0.00 ^b^

Data are given as mean values plus/minus standard deviation (n = 4). Different letters within the same column indicate significant differences (*p* < 0.05) between mean values.

**Table 5:** Sensory evaluation of surimi gel under three process conditions (0.5% and 1% salt).

Samples	Appearance	Aroma	Flavor	Texture	Overall Score
OS_1_	5.30 ± 0.86 ^ab^	4.95 ± 0.82 ^a^	5.10 ± 0.96 ^ab^	5.05 ± 0.82 ^bc^	5.41 ± 0.74 ^ab^
OSC_1_	4.95 ± 1.05 ^b^	4.95 ± 0.68 ^a^	4.95 ± 0.94 ^b^	4.85 ± 1.03 ^c^	4.84 ± 1.30 ^b^
TS_1_	4.95 ± 0.99 ^b^	4.80 ± 1.28 ^a^	5.05 ± 0.99 ^ab^	5.20 ± 1.00 ^bc^	5.46 ± 0.74 ^ab^
OS_2_	5.60 ± 0.82 ^a^	5.20 ± 1.32 ^a^	5.70 ± 0.86 ^a^	6.00 ± 0.64 ^a^	5.70 ± 0.92 ^a^
OSC_2_	5.05 ± 0.82 ^ab^	5.50 ± 0.76 ^a^	5.20 ± 1.15 ^ab^	5.55 ± 0.75 ^ab^	5.45 ± 0.60 ^ab^
TS_2_	5.50 ± 0.60 ^ab^	5.15 ± 0.93 ^a^	5.40 ± 0.68 ^ab^	5.45 ± 0.99 ^abc^	5.65 ± 1.22 ^a^

Data are given as mean values plus/minus standard deviation (n = 20). Different letters within the same column indicate significant differences (*p* < 0.05) between mean values.

**Table 6:** Factors and levels of surimi gel properties.

Level	Factors		
	Gelation Temperature (°C)	Gelation Time (h)	The Addition of PHP (%)
1	35.0	2.0	0.1
2	40.0	2.5	0.2
3	45.0	3.0	0.3

## Data Availability

Data will be made available on request.
